# Population Consequences of Physiological Responses to Stressors: A Road Map for Endocrine Data

**DOI:** 10.1093/icb/icag103

**Published:** 2026-07-11

**Authors:** Leslie New, Joshua Reed, Jennifer Jelincic, C Loren Buck, Enrico Pirotta, Kathleen Hunt

**Affiliations:** Department of Mathematics, Computer Science and Statistics, Ursinus College, Collegeville, PA, 19426, USA; Department of Mathematics, Computer Science and Statistics, Ursinus College, Collegeville, PA, 19426, USA; Marine Mammal Institute, Oregon State University, Newport, OR, 97365, USA; Department of Community, Environment and Policy, Mel & Enid Zuckerman College of Public Health, University of Arizona, Tucson, AZ, 85724, USA; Center for Research into Ecological and Environmental Monitoring, University of St Andrews, St Andrews, KY16 9LZ, Scotland; Marine Mammal Institute, Oregon State University, Newport, OR, 97365, USA

## Abstract

Environmental change causes physiological effects that can last years in long-lived species, yet there is a dearth of methods for quantifying such effects and their population consequences across the necessary decadal timescales. The Population Consequences of Multiple Stressors (PCoMS) framework is intended to link short-term responses to stressors to health, then to vital rates, and from there to population-level effects. To date, implementations of the PCoMS framework have primarily focused on behavioral responses of marine mammals to stressors, enabled by data available from biologging devices. However, technological advances have facilitated the collection of physiological data, particularly hormones, through techniques such as extraction and quantification of hormones in respiratory vapor (blow), feces, vibrissae, blubber, skin, and baleen, amongst others. Using baleen specimens from bowhead whales (*Balaena mysticetus*) that were sampled for stable isotopes and adrenal, thyroid, and reproductive hormones, we demonstrate how these individual histories can be used within the PCoMS framework to begin modeling the linkages between ecological change, anthropogenic actions, individual physiology, and potential population impacts.

## Introduction

Stressors are causal factors (physical, chemical, or biotic), either internal or external, that present a challenge to homeostasis, typically evoking various responses in individuals to help them mediate the stressor’s impact. These responses include the classic physiological “stress response,” a coordinated set of physiological changes that serve to limit energy spent on nonessential processes (e.g., growth, reproduction) and redirect energy toward coping with the stressor (Atkinson et al. 2015). While many of these responses are thought to be beneficial in the short-term, prolonged or repeated exposure to stressors can have long-term effects on individuals (Romero and Wingfield 2015). The physiological stress response is largely mediated via the endocrine system, with the glucocorticoid (GC) hormones playing a major role in affecting behavior, metabolism, reproduction, growth, and immune system modulation (Romero and Butler 2007). As a result, while responses to stressors occur at the individual level, there can be population-level consequences, particularly when a majority of the population (e.g., via environmental drivers) or a key demographic unit (e.g., breeding females) is exposed (Tyack et al. 2022). Further, stressors do not act in isolation, and multiple stressors can compound in ways that can cross physiological thresholds so as to produce unexpected effects.

Understanding the Population Consequences of Multiple Stressors (PCoMS) is a necessary component to assessing the influence of environmental drivers and anthropogenic actions on species of concern. A conceptual approach put forward by the National Academies of Sciences, Engineering and Medicine (NASEM), the PCoMS framework is intended to outline the mechanisms that link stressors to behavioral and physiological responses, health, vital rates, and resultant population dynamics (NASEM 2017). The ability to embed the PCoMS framework within a cumulative risk approach makes it applicable to multiple international management regimes, including the US, UK, EU, and Canada (Tyack et al. 2022). At the center of the PCoMS framework is the idea of individual health, defined as “the ability of an organism to adapt to and manage threats to survival and reproduction” (Tyack et al. 2022), and composed of indicators such as energy reserves and immune status (Pirotta et al. 2025b).

To date, most model implementations of the PCoMS framework (and its predecessor the population consequences of disturbance [PCoD] framework) have involved marine mammals, leading to a focus on species’ behavioral responses to stressors and the common usage of metrics of energy reserves to indicate health (e.g., New et al. 2014; Villegas-Amtmann et al. 2015; McHuron et al. 2017). Data limitations have driven this focus, as historically the deep-diving and wide-ranging nature of free-living marine mammal species has resulted in an emphasis on either remote data collection via biologging devices, or the collection of visual data such as behavior, photo-ID, and remote metrics of health (e.g., Kooyman 1985; Greller et al. 2020; Shero et al. 2021; Wright et al. 2021; Wiley et al. 2023). Some physiological data have been collected, often from in-depth health assessments and biopsy sampling (e.g., Barratclough et al. 2019; Gabriele et al. 2021), but historically these methods have lacked the temporal or contextual components required to inform the PCoMS framework. This is particularly true of the large whales, which, in contrast to pinnipeds and dolphins, cannot typically be physically handled except in situations in which they have stranded, limiting the opportunities for the collection of physiological data and ruling out hands-on health assessments.

While some observations of cetaceans’ underlying physiological processes can be collected remotely (e.g., size, visual health metrics [e.g., Bierlich et al. 2024; Robinson and Visona-Kelly 2025]), the endocrine data necessary to understand the physiological stress response have traditionally been elusive. However, the development of new technologies and field methods has facilitated the collection of endocrinological data from marine mammals via blood (e.g., Lidgard et al. 2008), blubber (e.g., Galligan et al. 2018), skin (e.g., Bechshoft et al. 2015), blow (e.g., Hogg et al. 2009), feces (e.g., Lemos et al. 2020), and baleen (e.g., Hunt et al. 2017). Often these analyses produce data on steroid hormones, which have the advantage of being evolutionarily conserved and readily detectable in most vertebrate tissues, while also including several hormones that play key roles either in the stress response itself (e.g., the GCs cortisol and corticosterone), or other axes that may be impacted by stress (e.g., reproductive hormones such as progesterone and testosterone and metabolic hormones such as thyroid hormones). Here, we outline how endocrinological data can be used to inform the PCoMS framework and present a case study of the bowhead whale (*Balaena mysticetus*), demonstrating how these data can inform the population consequences of physiological response to stressors not only for marine mammals, but for any species for which endocrine data are available.

### Population Consequences of Multiple Stressors framework

**Fig. 1:**
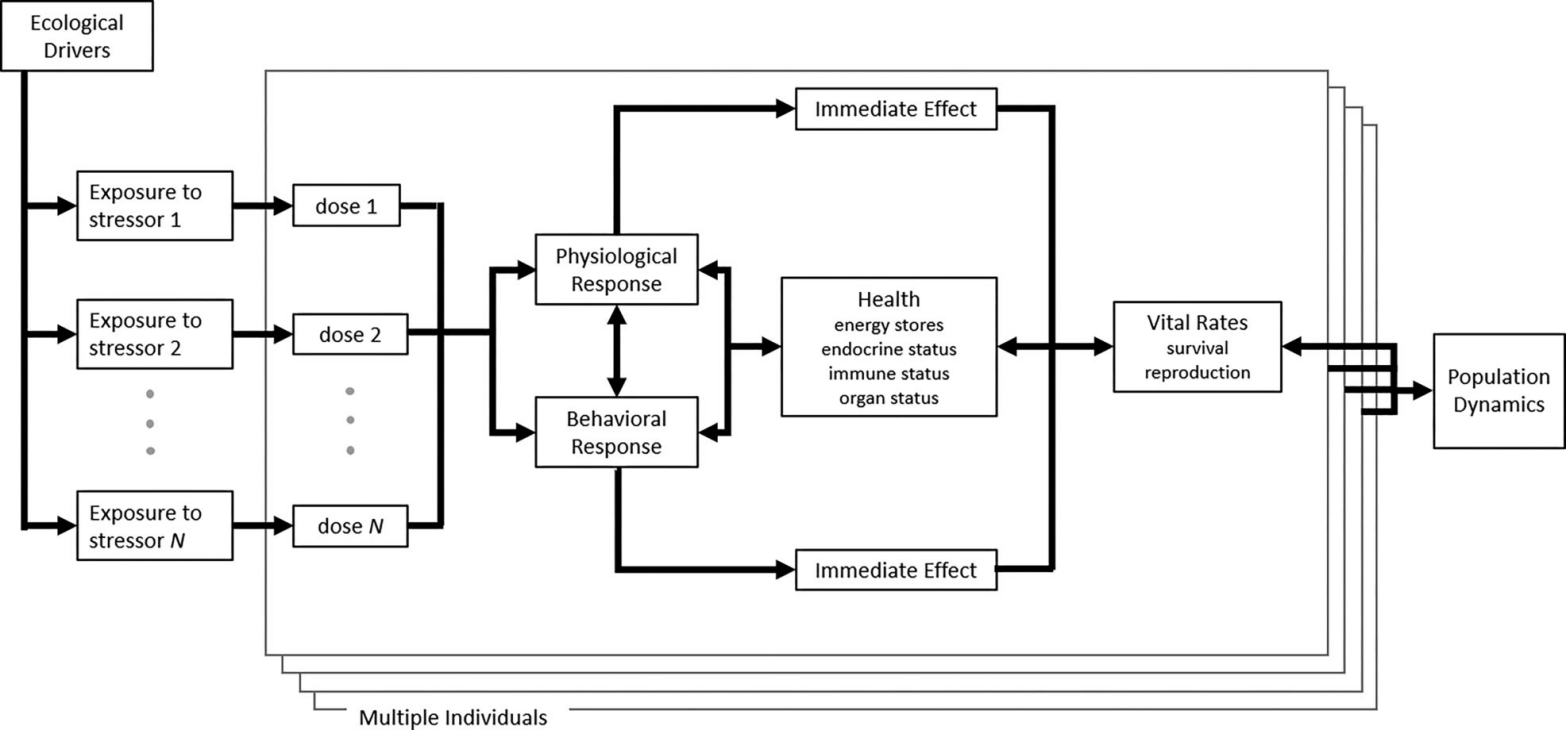
The population consequences of multiple stressors framework, modified from NASEM (2017) and Tyack et al. (2022). Each arrow represents a transfer function (i.e., statistical or process model) linking the different components of the framework. The stacked large boxes represent individuals, and it is the accumulated effect of the stressors across individuals that influences population dynamics.

The PCoMS framework provides a conceptual model by which the effect of a stressor on individual vital rates is mediated either directly by physiological and behavioral changes or else indirectly through health. Integrating the effects of a stressor over multiple exposed individuals can then give an indication of the effect on population dynamics, and thus the population-level consequences (Fig 1, NASEM 2017). The framework accommodates nonlinear pathways, allowing feedback between its different components. For example, exposure to a stressor may reduce survival, leading to a population decline, which in turn can result in increased fecundity as density-dependent pressures are removed.

The links between each step of the PCoMS framework are known as transfer functions, and can be formalized as statistical and process-based models that need to be informed by data and expert knowledge. Each transfer function allows the quantification of distinct phenomena that are important to increasing our understanding of biological phenomena, such as the physiological and behavioral changes resulting from exposure to a stressor (Pirotta et al. 2018). The inherent value in quantifying these links also means that PCoMS applications can build upon existing scientific knowledge on key biological relationships, incorporating mechanistic understanding of processes (e.g., New et al. 2014; Pirotta et al. 2022).

The data and scientific knowledge available are often the primary driver behind the choice of health indicator and pathway from stressor exposure to population consequence (Pirotta et al. 2018). For example, the first PCoD model, applied to southern elephant seals (*Mirounga leonina*), defined lipid mass as the metric of health, given the already established relationships between mother’s lipid mass and pup mass at weaning, which in turn influenced pup survival (Arnbom et al. 1993). The extensive biologging data available on this species then allowed for the estimation of changes in lipid mass over the course of foraging trips, permitting simulations exploring how changes in foraging behavior would affect health (New et al. 2014). As endocrinological data become more readily available for marine mammals, and the framework is extended to other species groups, it will become possible to explore stressor responses and measures of health, and links between the two, that have only previously been hypothesized as viable pathways.

### Physiological data for the PCoMS framework

PCoMS models are complex and data hungry, requiring long-term data sets and some knowledge of the underlying ecological mechanisms driving stressor response (Pirotta et al 2018). Endocrinological data that meet these criteria have been rare for marine mammals, originally primarily coming from feces (e.g., Lemos et al. 2020), while for a few species more in-depth health assessments are available (e.g., Barratclough et al. 2019). Respiratory vapor (blow) is emerging as a potential source of endocrinological data, although there are still difficulties in calibrating hormone concentrations (Burgess et al. 2018). As powerful as blow and feces are for informing aspects of marine mammal physiology, each sample is representative of a relatively short time scale and there can be logistical challenges in collecting the longitudinal and repeated sample data that can help inform the response of an individual to stressors. However, endocrine data can be extracted from linearly and continuously growing sample types such as whiskers (Keogh et al. 2021), teeth (Hudson et al. 2021) and baleen (Hunt et al. 2014) that accumulate hormones in a sequential fashion, creating a timeseries of biomarker data for a single individual that can cover timeframes ranging from weeks to years (e.g., Hunt et al. 2014, Hudson et al. 2021; Keogh et al. 2021).

These longitudinal endocrine profiles can be used to study key life history traits, such as reproduction (e.g., Hunt et al. 2016), as well as their relationship with factors such as stress (e.g., Crain et al 2021). For baleen whales, recent developments of methodology to extract hormone data from keratinized tissue, such as baleen, increases the number of species for which these profiles can be collected and enables the use of historical specimens archived at natural history museums (Hunt et al. 2014, 2026; Jelincic et al. 2026b). The timeframe these endocrine profiles represent can be determined by coupling endocrine measures with the analysis of stable isotope ratios, which can provide ecological context, such as differentiating among foraging habitats or identifying dietary shifts or changes in prey composition (Hobson and Welch 1992; Newsome et al. 2010). Further, stable isotope ratios fluctuate annually in many species, such that stable isotope analysis can clarify the timeline represented by the tissue. Thus, coupling stable isotopes with longitudinal hormone profiles can provide key insights into factors such as growth (e.g., Schell et al. 1989), reproduction (Hunt et al. 2022), or the duration and severity of disturbance events (e.g., Lysiak et al. 2018). The retrospective nature of longitudinal data from baleen provides perspective on past environmental and physiological conditions, and can be particularly useful for the provision of baseline data and when clear links can be established between past variability and current environmental or anthropogenic stressors.

Understanding long-term changes in individual endocrine profiles over time is important to conservation objectives (Hudson et al. 2021) and informing the PCoMS framework. However, sample types that represent endocrine responses over shorter timeframes, such as blood (e.g., McKenzie et al. 2004), blow (e.g., Hogg et al. 2009), urine (e.g., Lasley and Kirkpatrick 1991), feces (e.g., Rolland et al. 2012), and hair (e.g., Wilson et al. 2026) can still inform the transfer functions. Repeated measures from the same population contain valuable information, allowing comparison between populations experiencing different stressors (e.g., Barratclough et al. 2019), baseline data to help identify stress response (e.g., Rolland et al. 2012), and information on demography (e.g., Wilson et al. 2026) and reproductive seasonality (e.g., Fernández Ajo et al. 2025), amongst other important ecological processes.

In addition to stress responses, physiological data can provide metrics of health, a key component of the PCoMS framework. Visually assessed body condition metrics have been used as an indicator of health (e.g., Pirotta et al. 2025b; Schick et al. 2013), and may be used to represent an individual’s bioenergetic condition at a given time (Pirotta et al. 2025b), i.e. their nutritional state, although it is an imperfect proxy. The hormone triiodothyronine (T3) can also represent nutritional stress in many mammalian systems (Behringer et al. 2018), providing another potential measure of health. While bioenergetic models are common in applications of the PCoD framework (e.g., New et al. 2014; Villegas-Amtmann et al. 2015; McHuron et al. 2017), there are many potential health indicators in addition to nutritional state that can be informed by physiological data. Blood biomarkers have been used to infer inflammation and immune status (Weinstein et al. 2026), and respiratory exhalent microbiota are emerging as a potential metric for health (Miller et al. 2025). Moreover, physiological sampling can provide an indication of how stressor doses accumulate in an individual’s body before they manifest as an effect on health, e.g. toxicological reports informing individuals’ contaminant load (Bean et al. 2024).

### Fitting PCoMS transfer functions

Pirotta et al. (2018) provide a detailed overview of many of the analytical models used to implement the PCoD framework, and these methods remain relevant for PCoMS and to physiological data. No single approach will be suitable for every application, nor are there any that can inform all the transfer functions simultaneously. Instead, the analytical methods need to be appropriate to the data available and the question being asked. As a result, the same methods currently used to analyze physiological data outside the PCoMS framework will also be relevant to help inform the transfer functions.

The longitudinal hormone profiles available from tissues such as baleen (Hunt et al. 2014) and teeth (Hudson et al. 2021), require analysis of temporal patterns (i.e., cycles, seasonality, and trends over time) and there are many statistical techniques available to facilitate these analyses (Reed et al. 2026). Routine methods of summarizing hormone profiles, such as the iterative base-line approach and area under the curve (AUC) have been made more readily available to wildlife endocrinologists through the R package *hormLong* (Fanson and Fanson 2015). More classical time series approaches, such as autoregressive moving average and autoregressive integrated moving average can also be fit using general tools available in many software packages, including R (R Core Team, 2025), SAS (SAS Institute Inc. 2023), and SPSS (IBM Corp 2017).

Understanding the relationships between variables, such as how corticosterone (i.e., a proxy of physiological stress) covaries with testosterone (i.e., testosterone peaks indicating mating season) or δ^15^N (i.e., migration) (Hudson et al. 2025), is an important component of the PCoMS framework, and increases our understanding of physiological processes more broadly. Cross-correlation analysis, which measures the relationship between two timeseries as a function of the relative lag between them (Cryer and Chan 2008), can be used to explore these associations, as can regression methods that explicitly account for the autocorrelation in the residuals that occurs when a timeseries is the response variable (e.g., Jelincic et al. 2026b, Mitchell et al. 2020). More complex methods, such as state-space models, can also be used when faced with issues such as combining multiple data sources, missing or irregularly spaced data, and an interest in latent, unobservable states (e.g., true underlying nutritional and stress state) (e.g., Pirotta et al. 2025a, 2025b). Simple comparisons of endocrine data in the presence and absence of stressors can be done with nonparametric tests (e.g., Rolland et al. 2012). Transfer functions between stressors and responses can also be formalized into dose–response functions, in which the probability of a physiological change is modeled as a function of the received dose of a stressor; e.g. assessing the relationship between fecal GC concentrations and individual exposure to different stressor intensities (Pirotta et al. 2023). Many of the PCoMS transfer functions are centered on basic and applied ecological relationships, and dose–response functions provide a fundamental tool for these assessments (Rosenfeld et al. 2022). Lastly, classification methods, such as random forests, can be used to predict demographic parameters (e.g., Wilson et al. 2026), which can help define population structure. Given that certain demographic groups may be more sensitive to stressors (e.g., pregnant females, Villegas-Amtmann et al. 2015), information regarding the population structure is particularly valuable for the final transfer function linking vital rates to population dynamics.

The connection between vital rates and population dynamics is probably one of the best understood in ecology. Tools such as matrix population models and population viability analysis work on the population level, accounting for the population’s demographics through either age- or stage-structured models, often conceptualize the change in a population through time in discrete steps (e.g., Caswell 2001; Lacy 2018), and have been used within the PCoD framework (e.g., Pirotta et al. 2025b; New et al. 2014). However, given that the PCoMS (and PCoD) framework scales individual response to stressors to population-level effects, individual-based models (IBMs) (Grimm and Railsback 2013), sometimes referred to as agent-based models, are a particularly powerful tool in this context (e.g., Nabe-Nielsen et al. 2018). IBMs are computer simulations that work at the scale of individual organisms, each of which can have its own attributes (e.g., demographic group and health), behaviors (e.g., growth, reproduction, and movement), and physiological state (e.g., size and endocrine status). This means that population dynamics arise from individual processes (e.g., response to stressors) and the interactions of individuals with one another and the environment.

### Bowhead Whale Case study

**Fig. 2:**
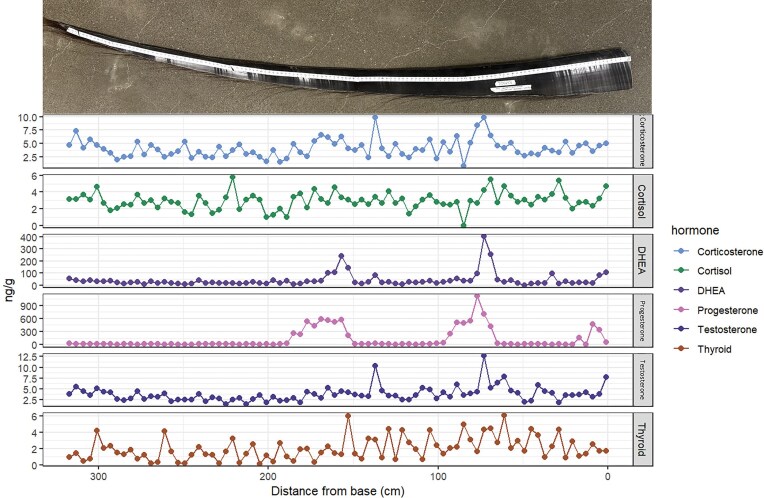
Top: A single bowhead whale baleen plate showing measuring tape (affixed with 0 cm = proximal-most point of base of plate, on right) and typical sampling grooves drilled every 1 cm. (photo credit: Jennifer Jelincic) Bottom: Female bowhead hormone profile presenting six hormones (cortisol, corticosterone, DHEA(S), progesterone, testosterone, and T3). The x-axis displays the timeseries as the distance from the base of the baleen plate, with the proximal-most point on the right, representing the most recent hormone measurements.

Bowhead whales are a high-latitude mysticete that migrates between arctic summer forging grounds and subarctic wintering grounds, following the moving border of the pack ice. We focus here on the largest population of bowhead whales, the Bering–Chukchi–Beaufort population, which migrates between the Arctic Ocean in the summer and the North Pacific/Bering Sea in the winter (George and Thewissen 2020). As a result, this population is exposed to ecological oscillations known to happen in the North Pacific (Mantua and Hare 2002; Overland and Stabeno 2004; Newman et al. 2016), as well as the rapid environmental change characteristic of the modern Arctic. Given that bowhead whales have some of the largest baleen and were the target of commercial whaling as well as on-going Native hunts, there is a rich archive of keratinized baleen specimens available from museums that can be used to build individual endocrine profiles covering up to 20 years of the individuals’ lifespan (e.g., Hunt et al. 2022; Jelincic et al. 2026; Fig. 2). Although baleen has limited availability even within mysticetes, existing tissue and sample types for other species, such as whiskers in pinnipeds (Keogh et al. 2021) and feces in gray whales (*Eschrichtus robustus*, Pirotta et al. 2023), provide analogous data, making bowhead whales a valuable case study. Our goal is not to fully implement an application of the PCoMS framework, but instead to use the bowhead whale as an example to illustrate how certain types of data can, or cannot, serve to populate the relevant transfer functions, what sort of information may be needed about the species, and how various types of data gaps can be addressed and overcome.

**Fig. 3:**
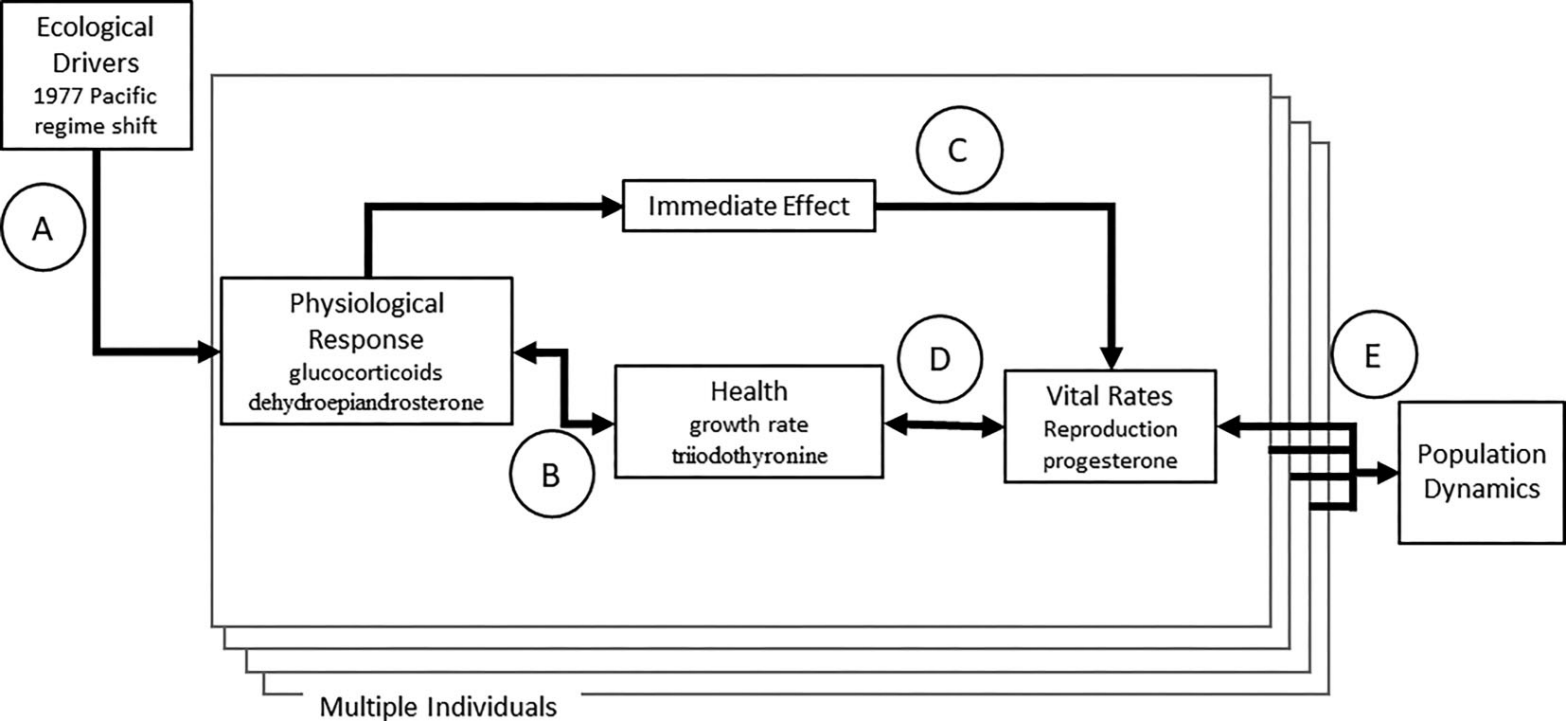
The PCoMS framework for bowhead whales, with the individual transfer functions lettered to facilitate discussion within the text.

To explore bowhead whales’ response to environmental change, we accessed museum specimens collected before or after the 1977 regime shift in the North Pacific and Bering Sea. This well-documented regime shift coincided with population declines of many marine mammals, seabirds, and fish in the years that followed (Hare and Mantua 2000). From these baleen plates, we subsampled baleen powder at regularly spaced intervals along the length of the tissue. Subsamples were extracted and quantified for four hormones that may be influenced by acute or chronic stress, cortisol, corticosterone, dehydroepiandrosterone (DHEA), triiodothyronine (T3), as well as two reproductive hormones, progesterone and testosterone. Subsamples were further analyzed for stable isotopes of carbon and nitrogen (Jelincic et al. 2026a, 2026b). Metadata on each individual included information (if known) such as sex, body length at death, and date of death. The use of these endocrine profiles enabled exploration of the physiological consequences of stressors on individual health and reproduction. However, it is the specific hormones sampled, coupled with information from the scientific literature, that are used to define the nature of the transfer functions and the indicator used for health. Together, these define the PCoMS framework linking bowhead whale response to environmental drivers to changes in population dynamics (Fig. 3).

The first transfer function (Fig. 3A) links ecological drivers to physiological change. Given the abrupt nature of the regime shift and the fact that it affected all individuals in the population, the effect of the stressor can be assessed by comparing the distribution of hormone concentrations in individuals before and after 1977. Given the documented negative impacts of the regime shift on many species including the bowhead whales (e.g., Hare and Mantua 2000), we expect that the individuals post-1977 would experience more chronic stress in comparison to before the regime shift, and that this change would be reflected in the endocrine profiles.

The second transfer function (Fig. 3B) links physiology to individual health. The 1977 regime shift is thought to have resulted in reduced prey availability for certain species, and can be hypothesized to have caused nutritional stress in adult whales as well as juveniles. As nutritional stress reduces energy reserves and hence affects health, a biomarker of nutritional stress can be monitored via the thyroid hormone T3, which classically reduces in starvation (an adaptive response that serves to reduce metabolic rate) (e.g., Groscolas and Leloup 1989). However, in whales, T3 does not always decline as expected during nutritional stress (Lysiak et al. 2018; Ajó et al. 2024), possibly related to the fact that starvation in whales also involves thinning of the insulative blubber layer, with complex and poorly understood effects on metabolic rate. Therefore, while the second transfer function can potentially be informed by the relationship between GCs and T3, it remains to be determined whether T3 data can serve this purpose for cetaceans.

An alternative method to assess the link from stress to health, however, is assessment of growth rate. Chronic stress (especially chronic nutritional stress) in juvenile mammals, as well as their parents, decreases growth rate (Stewart et al. 2021), which can reduce adult body size with consequences for reproductive success (Romero and Wingfield 2015; Pirotta et al. 2024). Juvenile bowheads display rapid baleen growth as well as rapid body length growth in their first year, during which they are nursing from their mothers. However, after weaning (at approximately 10 months of age), they experience a near cessation in body growth, and for the next three to four years, only the head and baleen continue to grow (George et al. 2016). Thus, existing natural history data can provide confidence that stress and growth do have the hypothesized relationships for a given study species. If such is a case, a logical approach to link individual physiological responses to individual health is to examine relationships between stress biomarkers and growth metrics (Fig 3B). For example, for bowheads, one potential approach is to assess the relationships between a juvenile’s lifelong body-length growth rate, as well as its baleen growth rate, and a panel of hormones that together may indicate stress (cortisol, corticosterone, DHEA, and T3), both before and after 1977.

In cases of data gaps, PCoMS modeling approaches can often accommodate inferential approaches or proxies in lieu of more direct measurements. In the bowhead example, direct measurements of lifelong growth rate do not exist for individual juveniles. However, average lifetime growth rate can be inferred if the age of the individual can be estimated, and age estimation techniques do exist for many taxa, juvenile bowheads included. For example, the baleen of young bowhead whales is representative of the individual’s entire life span (as long as the “natal notch” has not yet begun to erode away; see Fernández Ajó et al. 2020). This allows for the calculation of a known age at death based upon the number of annual stable isotope cycles (Schell et al. 1989; Lubetkin et al. 2008), and, when coupled with the body length at death, can be used to calculate the average lifelong growth rate (body length/age). Furthermore, the width of each stable isotope cycle provides age-specific annual baleen growth rate, providing further insight into nutritional stress of certain specific years. Together with the hormone profiles, these data enable the assessment of the potential negative association between GCs (i.e., stress) and growth.

Though juveniles’ response to chronic environmental stress may be to reduce growth, adults cannot respond in the same way. Instead, the classic response of adult vertebrates to nutritional stress is to suspend or delay reproduction (Romero and Wingfield 2015). Therefore, even in the absence of such a growth-reduction signal in adults, a direct link between physiological change and vital rates (Fig. 3C) can be explored, given the established relationships between elevations in GCs and disruptions in reproduction (Romero and Wingfield 2015). Endocrine data of many mammals can provide information on these effects on reproduction, particularly the steroid hormones progesterone in females, and testosterone in males. In our bowhead example, progesterone patterns in baleen of adult females reveal recent pregnancies and intercalving intervals, and the occurrence and regularity of testosterone cycles in males provide further information on timing and regularity of breeding efforts, in relation to prior or simultaneous stressors.

Changes in juvenile growth can also be tied to subsequent reproduction, informing the transfer function linking health and vital rates (Fig. 3D). Knowing whether a present-day adult experienced the 1977 regime shift while a juvenile would be informative for this transfer function, especially in combination with data on its body length and reproductive history. However, given the long lifespan of bowhead whales (>200 years) (George et al. 1999), and the fact that the baleen only captures at most the last 20 years of an individual’s life, baleen alone cannot be used to determine if an adult individual caught in more recent years was born before or after the regime shift. Techniques now do exist for determining age of adult bowheads, e.g. by racemization of aspartic acid in the eye lens (e.g., George et al. 1999; Rosa et al. 2013) or epigenetics (Zoller et al. 2025). Though eye lenses were not routinely collected in the past, future datasets may provide more information on the age of a given adult and hence whether it experienced unusual ecological pressures while a juvenile. Further, lifetime reproduction in female bowheads, as in many mammals, can be estimated via counting of *Corpea albicantia* (representing ovulation events) in the ovaries. Therefore, though the data available at present for this species cannot inform the transfer function connecting health and vital rates (Fig. 3D), this link may be addressable in the future. Further, the scientific literature allows us to inform this link using the established relationship connecting growth to reproductive success and survival in other mammals (e.g., Lee et al. 2012; Marcil-Ferland et al. 2013; Pirotta et al. 2025a).

The first four transfer functions in the PCoMS framework capture the effects of stressors on individuals. Therefore, the last transfer function needs to integrate the effect of stressors across multiple individuals to link changes in vital rates to population dynamics (Fig. 3E). In this case, this last step can be achieved using an IBM (Grimm and Railsback 2013). This works by first simulating individual GC profiles according to stressor exposure and age–sex classification. The simulated data are then used as the input into the models established above that connect GC to growth, and growth to reproduction, for juveniles, and that connect GC to reproduction for adults. All individuals are simulated to be affected by environmental change, given the scale of the 1977 regime shift, and the effect is likely to be relatively consistent across all members of the population (e.g., same percentage reduction in prey, although baseline availability may vary with location), so there is no need to determine the proportion of the population affected or differing levels of exposure, as is necessary in other PCoMS applications (Pirotta et al. 2018; Tyack et al. 2022). Simulating GC profiles directly from the observed data allows us to investigate the observed influence of the historical regime shift on the bowhead whale population. However, by sampling only from the more extreme values for the GC response to stressors, or even from hypothesized, more severe responses than observed, it would be also possible to explore potential changes to a population’s trajectory in response to increasingly severe environmental change.

## Conclusions

The physiological pathway linking exposure to stressors, response and population consequences has been part of the PCoMS framework, and its predecessors, almost from the beginning. However, the difficulty in collecting longitudinal physiological data from free-living marine mammal species has previously limited the ability to explore these connections, despite physiological effects being the fundamental route by which ecological change affects vital rates. The stress response is thought to be the proximate cause of most stressor-related behavioral changes, which at extreme values trigger an “emergency life history state” (Wingfield et al. 1998) that can change reproductive and foraging behavior—the very behaviors that until recently were the main focus of the PCoMS framework. Our example of timeseries endocrine data derived from whale baleen provides a demonstration of how endocrine analysis can inform the links between individual physiological response to stressors and health, as well as insights into potential long-term effects of environmental change on population dynamics. Bowhead whales are only one example; the details of the bowhead whale system presented above serve as an illustration of how the model’s transfer functions can be approached given data type, availability, and limitations, as well as how certain data gaps can be overcome. Importantly, the data types discussed here are often available for terrestrial species, which also permit the exploration of testable hypotheses for the combined effects of multiple stressors (e.g., Wilsterman et al. 2020), highlighting avenues for the greater application of the PCoMS framework to species other than marine mammals.

Each step of the PCoMS framework provides important ecological insights in its own right, such as the link between stress state and reproduction (e.g., Pirotta et al. 2025a) or the variation in hormone concentrations across periods of different stressors (Jelincic et al. 2026). These steps are more often than not the focus of studies independently of the PCoMS framework (e.g., Arnbom et al. 1993; Frid and Dill 2002; Bender et al. 2007; Jesmer et al. 2017). The explicit building blocks of the PCoMS framework is to bring these individual pieces together, helping us understand a bigger picture that is greater than the sum of its parts. Thus, PCoMS provides the opportunity to explore ecological theory, the influence of stressors, and the management and conservation needs of many species.

## Author contributions

L.N.: Conceptualization, funding acquisition, investigation, methodology, visualization, writing—original draft, JR: Investigation, methodology, writing—review & editing, J.J.: Investigation, methodology, wiring—review & editing, CLB: Conceptualization, funding acquisition, investigation, writing—review & editing, E.P.: Methodology, writing—review & editing, KH: Conceptualization, funding acquisition, investigation, visualization, writing—review & editing.

## Data Availability

Not applicable.

## Notes

From the symposium “Hormones at sea: New frontiers in marine mammal endocrinology and ecophysiology” presented at the annual meeting of the Society for Integrative and Comparative Biology, January 3–7th, 2026.
